# Methylglyoxal, a highly reactive dicarbonyl compound, as a threat for blood brain barrier integrity

**DOI:** 10.1186/s12987-023-00477-6

**Published:** 2023-10-24

**Authors:** Eline Berends, Robert J van Oostenbrugge, Sébastien Foulquier, Casper G Schalkwijk

**Affiliations:** 1 Department of Internal Medicine, Maastricht University, Universiteitssingel, Maastricht, 50 6229ER The Netherlands; 2 Cardiovascular Research Institute Maastricht (CARIM), Maastricht University, Universiteitssingel 50, Maastricht, 6229ER The Netherlands; 3 School for Mental Health and Neuroscience (MHeNs), Maastricht University, Universiteitssingel 40, Maastricht, 6229ER The Netherlands; 4 Department of Neurology, Maastricht University Medical Centre (MUMC+), P. Debyelaan 25 6202AZ, Maastricht, The Netherlands; 5 Department of Pharmacology and Toxicology, Maastricht University, Universiteitssingel 50 6229ER, Maastricht, The Netherlands

**Keywords:** Glycation, Neurovascular unit, Brain, Advanced glycation end-products, Endothelial cells, Astrocytes, Microglia, Pericytes, Vascular smooth muscle cells

## Abstract

The brain is a highly metabolically active organ requiring a large amount of glucose. Methylglyoxal (MGO), a by-product of glucose metabolism, is known to be involved in microvascular dysfunction and is associated with reduced cognitive function. Maintenance of the blood-brain barrier (BBB) is essential to maintain optimal brain function and a large amount of evidence indicates negative effects of MGO on BBB integrity. In this review, we summarized the current literature on the effect of MGO on the different cell types forming the BBB. BBB damage by MGO most likely occurs in brain endothelial cells and mural cells, while astrocytes are most resistant to MGO. Microglia on the other hand appear to be not directly influenced by MGO but rather produce MGO upon activation. Although there is clear evidence that MGO affects components of the BBB, the impact of MGO on the BBB as a multicellular system warrants further investigation. Diminishing MGO stress can potentially form the basis for new treatment strategies for maintaining optimal brain function.

## Background

The brain is a highly metabolic organ, with glucose as its predominant energy source [1, 2]. To supply the brain with sufficient glucose, as well as nutrients and oxygen, a high vascular perfusion is essential [3]. On the other hand, the brain is immune privileged [4, 5]. The fine balance between the supply of essential compounds, such as glucose, and the protection of the brain against toxins and pathogens, is maintained by the blood-brain barrier (BBB) [6]. Maintenance of the BBB is therefore essential to maintain optimal brain function. Although the concept of immune privilege of the central nervous system in its classical definition has been debated, it is well established that the unique features of the brain’s immune system, require protection against toxins and pathogens [7–9]. A large body of evidence indicates that BBB integrity is impaired in neurodegenerative diseases like multiple sclerosis, Alzheimer’s disease, epilepsy, and cerebral small vessel disease [10–12], but also in hypertension [13] and diabetes [14].

**Fig. 1:**
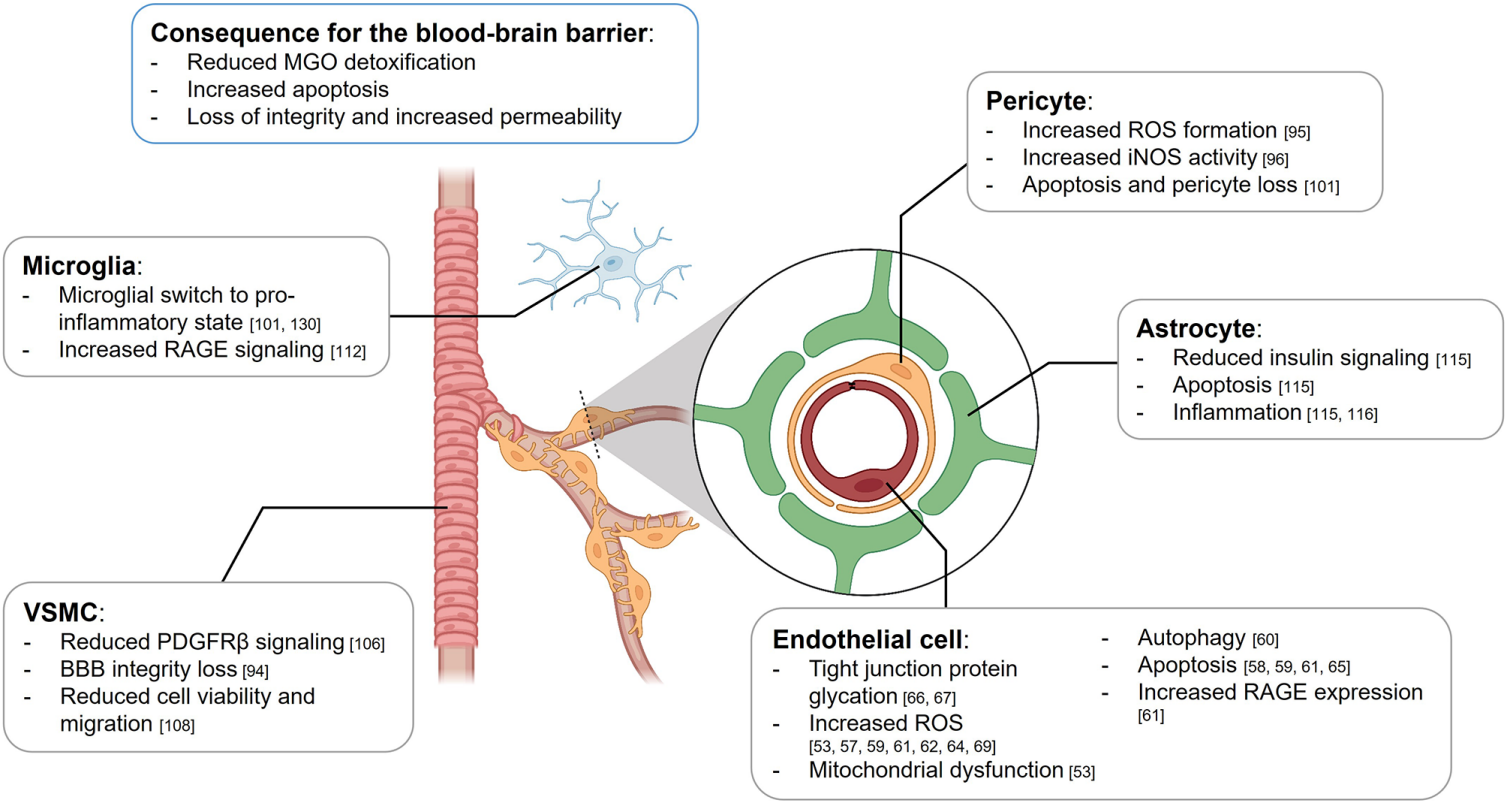
Effects of methylglyoxal (MGO) on the different cell types of the blood-brain barrier (BBB). Schematic representation of the BBB including vascular smooth muscle cells (VSMCs), pericytes, endothelial cells, astrocytes, and microglial cells. The boxes contain the effect MGO exposure has on the respective cell type including the cited literature. Abbreviations: reactive oxygen species (ROS); inducible nitric oxide synthase (iNOS); platelet-derived growth factor receptor β (PDGFRβ); receptor of advanced glycation end products (RAGE)

The BBB has a complex structure that involves multiple cell types, each playing a different yet crucial role in the regulation of the barrier function [15–18] (Fig. 1). Endothelial cells form the brain microvessels with a high expression of tight junction proteins preventing metabolites from flowing passively from the blood into the brain [16], thereby functioning as a first barrier. Larger cerebral vessels are surrounded by mural cells, including vascular smooth muscle cells (VSMCs) around the larger vessels, which transition to pericytes while reaching the capillary bed, with a notable high ratio of pericytes to endothelial cells [10, 18]. The vessels in the brain containing endothelial cells and mural cells, are surrounded by astrocyte endfeet, the glia limitans, forming the perivascular space and a second barrier between the blood and brain [5, 19]. The BBB is surveyed by microglial cells, the resident immune cell of the brain [20]. The presence of the BBB makes cerebral microvessels structurally and functionally different from microvessels elsewhere in the body [21].

While glucose is essential for the brain as energy source, a downside of glycolysis is the production of (toxic) glycolysis by-products [22]. A well-studied toxic by-product of glycolysis is methylglyoxal (MGO), a risk factor for microvascular complications. MGO is known to be increased in endothelial cells during hyperglycemic events due to the insulin-independent uptake of glucose [22]. Likewise, glucose transport across the BBB is also predominantly through insulin-independent glucose transporters [23]. Therefore, an acute increase in glucose in the circulation leads directly to an increase in glucose transport across the BBB, in turn leading to an increase in glycolysis and MGO formation in the brain [2, 24].

Although there is a wide range of data available on the toxic effects of MGO on different compartments of the BBB, a detailed description of the effect of MGO on the BBB is missing to date. In this review, we give a brief overview of what MGO is, followed by an overview of the effect of MGO on different BBB cell types, including endothelial cells, mural cells, astrocytes, and microglia.

## Methylglyoxal

### Formation, detoxification, and clearance of methylglyoxal

**Fig. 2:**
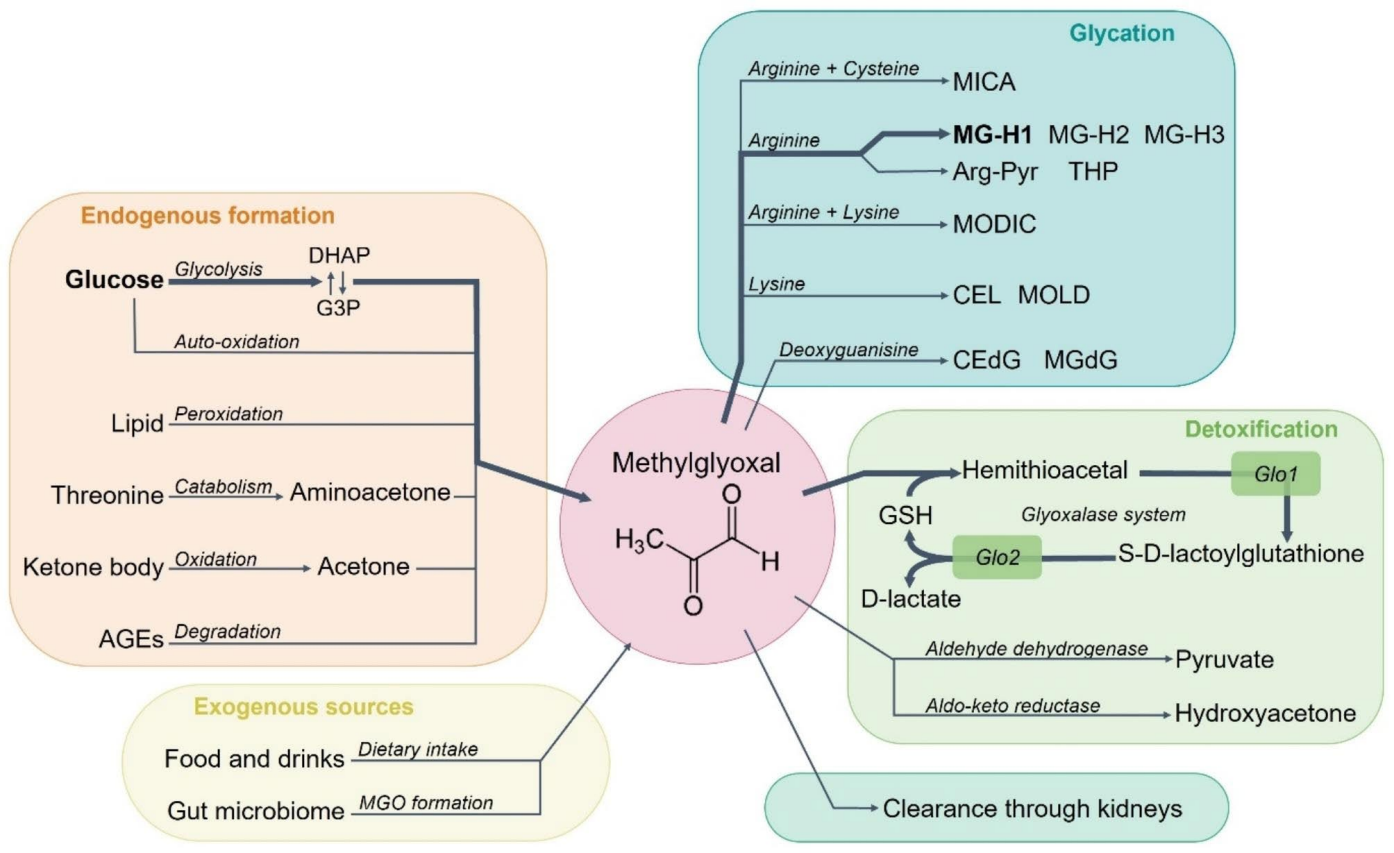
Formation, glycation and detoxification of methylglyoxal (MGO). MGO can be formed endogenously from glucose through auto-oxidation [26], or spontaneous degradation of dihydroxyacetone phosphate (DHAP) and glyceraldehyde-3-phosphate (G3P) during glycolysis [25]. Additionally, MGO can be formed through lipid peroxidation [27], threonine catabolism [28], ketone body oxidation [29], and the degradation of advanced glycation end-products (AGEs) [26]. Moreover, MGO can be increased in a system through exogenous sources in the form of dietary intake [22] and local formation by the gut microbiome [31–33]. MGO can glycate to form AGEs. These include glycation with arginine forming hydroimidazolones (MG-H1, MG-H2 and MG-H3), Nδ-(5-hydroxy-4,6-dimethylpyrimidine-2-yl)-L-ornithine argpyrimidine (Arg-Pyr) or Nδ-(4-carboxy-4,6-dimethyl-5,6-dihydroxyt-1,4,5,6-tetrahydropyrimine-2-yl)-L-ornithine (THP) [22, 38, 40]. MGO glycation with lysine forms N^ε^-(1-carboxyethyl)lysine (CEL) or the lysine dimer 1,3-di(N^ε^-lysino)-4-methyl-imidazolium (MOLD) [41]. Crosslinking between arginine and cysteine leads to the formation of mercaptomethylimidazole crosslinks between arginine and cysteine (MICA) [43]. Crosslinking between arginine and lysine results in 2-ammonio-6-((2-[(4-ammonio-5-oxido-5-oxopentyl)amino]-4-methyl-4,5-dihydro-1H-imidazol-5-ylidene)amino)hexanoate (MODIC) [42]. DNA glycation can occur between MGO and deoxyguanosine resulting in products N2-carboxyethyl-2’-deoxyguanosine (CEdG) and 3-(2’-deoxyribosyl-6,7-dihydro-6,7-dihydroxy-6/7-methylimidazo-[2,3-b]purin-9(8)one (MGdG) [45]. MGO can be detoxified into D-lactate through the glyoxalase system entailing glyoxalase 1 (Glo1) and glyoxalase 2 (Glo2), with glutathione (GSH) as a co-factor [37, 38]. Additionally, minor detoxification pathways exist, such as aldehyde dehydrogenase and aldo-keto reductase [22]. Lastly, MGO can be cleared through the kidneys without any detoxification or glycation [22]

MGO is a highly reactive dicarbonyl compound that can be formed endogenously through several different pathways (Fig. 2). MGO is predominantly formed by spontaneous degradation of dihydroxyacetone phosphate (DHAP) and glyceraldehyde-3-phosphate (G3P), which form during glycolysis [22, 25]. While glycolysis is estimated to account for 90% of endogenously formed MGO [22], MGO can also be generated in relatively smaller amounts during auto-oxidation of glucose [26], lipid peroxidation [27], oxidation of aminoacetone in threonine catabolism [28], and oxidation of acetone in ketone body metabolism during diabetic ketoacidosis [29]. Additionally, MGO can be formed during enzymatic degradation of glycated proteins [26].

The digestive system can take up MGO from exogenous sources, which include dietary MGO, and MGO formed by the gut microbiome. Dietary intake of MGO can increase plasma MGO levels, although the amount of exogenous MGO contributing to increased levels of circulating MGO is believed to be very small [22]. The main contributors to dietary intake of MGO are heat-treated products including baked goods, fish, meat, nuts, and coffee [30]. Additionally, several types of bacteria in the gastro-intestinal tract express MGO synthase and have been shown to produce MGO [31–33]. The exact contribution of MGO produced by the gut microbiome, to circulating MGO levels, is difficult to estimate as the accumulation of MGO produced by bacteria is known to be low [32] and because MGO is likely to react with the high amount of protein present in the intestinal tract [34–36].

MGO can be cleared through detoxification or elimination [22]. Under physiological conditions, MGO is predominantly detoxified through the glyoxalase system [22, 37]. Within this system the rate limiting enzyme glyoxalase 1 (Glo1) converts MGO together with cofactor glutathione (GSH) into S-D-lactoylglutathione, followed by conversion into D-lactate by glyoxalase 2 (Glo2) [38]. Other minor detoxification pathways are aldehyde dehydrogenase (ALDH) which catalyzes the oxidation of MGO forming pyruvate, and aldo-keto reductase (AKR) which metabolizes MGO into hydroxy-acetone [22]. The body can additionally clear MGO through the kidneys without prior detoxification [22].

### Methylglyoxal reactivity and glycating capacities

MGO is a highly reactive molecule, which is thousands of times more reactive than glucose. MGO can react with proteins, DNA, and lipids, leading to functional alterations of these compounds [22, 26, 38]. The irreversible reaction of MGO with protein leads to the formation of advanced glycation end-products (AGEs) and occurs predominantly with nitrogen-rich amino acids, such as arginine, and to a smaller extent, lysine [39]. The reaction of MGO with arginine will lead to the formation of hydroimidazolones (MG-H1, MG-H2 and MG-H3), *Nδ*-(5-hydroxy-4,6-dimethylpyrimidine-2-yl)-L-ornithine argpyrimidine (Arg-Pyr) or *Nδ*-(4-carboxy-4,6-dimethyl-5,6-dihydroxyt-1,4,5,6-tetrahydropyrimine-2-yl)-L-ornithine (THP) [22, 38, 40]. The reaction of MGO with lysine residues will result in formation of MGO-derived AGEs *N*^*ε*^-(1-carboxyethyl)lysine (CEL) or the lysine dimer 1,3-di(*N*^*ε*^-lysino)-4-methyl-imidazolium (MOLD) [41]. MGO can also react with lysine and arginine residues with the formation of the crosslink 2-ammonio-6-((2-[(4-ammonio-5-oxido-5-oxopentyl)amino]-4-methyl-4,5-dihydro-1 H-imidazol-5-ylidene)amino)hexanoate (MODIC) [42].

Recent evidence showed that MGO can also form stable mercaptomethylimidazole crosslinks between arginine and cysteine (MICA) in proteins [43]. MICA modifications can occur within or between proteins. It was shown that this type of modification can form dimers of Kelch-like ECH-associated protein 1 (KEAP1) proteins, which in turn can activate nuclear factor erythroid 2-related factor 2 (Nrf2) transcription factor, and thereby promote the transcription of antioxidant genes [44]. This would suggest a function as a post-translation protein modification in response to glycolytic stress [44].

The binding of MGO with deoxyguanosine leads to the formation of DNA glycation products *N*^*2*^-carboxyethyl-2’-deoxyguanosine (CEdG) and 3-(2’-deoxyribosyl-6,7-dihydro-6,7-dihydroxy-6/7-methylimidazo-[2,3-b]purin-9(8)one (MGdG) [45]. These modifications are seen as DNA damage as a response to methylglyoxal stress, however, the subsequent effects are currently unclear [22].

### Methylglyoxal-associated neuropathologies

Under pathological conditions, such as diabetes, MGO accumulation can increase due to a higher formation, a reduction in its detoxification by the glyoxalase system which becomes exhausted and dysfunctional, and due to a reduction in its clearance subsequent to a reduced kidney filtration. Consequently, this leads to an increase in MGO-derived AGE formation and DNA glycation. This elevated methylglyoxal stress and glycation have been shown to lead to endothelial dysfunction, micro- and macrovascular dysfunction, pancreatic beta-cell dysfunction, cancer, neurodegeneration, and cognitive disorders, as extensively reviewed earlier [22].

There is an increasing number of studies showing the association between neurological disorders and cognitive function and AGEs [46]. Elevated dicarbonyls in plasma and hyperglycemia, are associated with occurrence of cardiovascular incident, including stroke [47], and predicts a worsened outcome in stroke [48]. Moreover, the amount of AGEs in the brain and CSF increases with age, and is more prevalent in people with Alzheimer’s disease [49, 50] and multiple sclerosis [51]. MGO has also been linked to the prevalence and treatment of psychiatric disorders, with both positive and negative effects on brain function [52]. Few studies in animals have shown a clear mechanistic link between MGO and AGEs and the pathological effects it has on the brain. MGO has been shown to be involved in BBB integrity loss in a model for type 1 diabetes in rats [53], and reduction of MGO reduces infarct size after induction of ischemic stroke in mice [54]. Since most results are based on associations, it is difficult to conclude a causal link between MGO and cognitive impairment.

We propose here that the mechanism by which MGO causes cognitive impairment is through microvascular impairment and BBB integrity loss. In this review, we summarize what is currently known about MGO and its effects on the different cell types of the BBB, starting with brain microvascular endothelial cells.

## The toxicity of methylglyoxal in brain microvascular endothelial cells

Substantial data is showing a role of MGO in endothelial dysfunction and negative consequences for peripheral and retinal microcirculation [22, 55]. Nonetheless, due to the distinctive phenotype of brain microvascular endothelial cells (BMECs), it is important to take a closer look into the effect of MGO on endothelial cells in the brain specifically.

Several studies have shown harmful effects of exogenous MGO on BMECs in vitro. MGO induces a reduced cell viability of BMECs [53, 56–64], causes cell membrane damage [60, 64] alters morphology [62], and increases apoptosis [58, 59, 61, 65] and cell death [57–59, 61, 62]. These biological effects of MGO may reduce the barrier function of BMECs. Indeed, in vitro BBB functional assays with a human BMEC cell line have revealed a decreased trans-endothelial electrical resistance (TEER) upon MGO treatment after 4 h with concentrations of ≥ 600μM MGO, reflecting an increased endothelial barrier permeability [62, 66, 67]. This has been confirmed in permeability assays using fluorescent tracers, showing an MGO-induced increase in BMEC permeability for different particle sizes (390 Da to 150 kDa) [57, 62, 66].

Evidence for a role of MGO and MGO-derived AGEs in BMEC barrier permeability has also arisen from in vitro studies with aminoguanidine, a well-studied scavenger of MGO [68]. MGO causes an increase in MGO-derived AGEs in BMECs [58, 59, 61] and co-treatment of BMECs with aminoguanidine results in reduced MGO-derived AGE formation [58, 61] and subsequently prevents MGO-mediated reduction in viability [61] and prevented changes in endothelial permeability [58, 61, 62]. Although evidence supporting an effect of MGO on BMECs in vivo is lacking, a robust set of data shows that MGO leads to an impairment of BMECs function and subsequently BBB dysfunction.

### Glycation of tight junction proteins by methylglyoxal

The unique high expression of tight junction proteins in BMECs is important for ensuring the impermeability of the BBB [16]. MGO treatment of BMECs alters gene expression patterns of transporter proteins and tight junction proteins [57]. Gene expression of claudin-5 and occludin, which are the highest prevalent transmembrane tight junction proteins in the BBB, were reduced as consequence of MGO treatment [53, 57]. Reduced claudin-5 expression leads to a greater barrier permeability, and reduced occludin expression is known to alter calcium fluxes into the brain, both reducing BBB integrity [10].

Additionally, several studies have demonstrated an MGO-induced change in tight junction protein localization. In BMECs incubated with MGO, β-catenin and claudin-5 localization was less pronounced at the cell-cell junctions, which was normalized when co-treated with aminoguanidine [57, 62]. MGO treatment also showed to alter the localization of zonula occludens 1 (ZO-1) [66], which is a major tight junction-associated protein that locks transmembrane tight junction proteins to the cell’s cytoskeleton [10]. The MGO-induced ZO-1 localization was not observed when the cells were co-treated with N-acetyl cysteine (NAC), a direct quencher of MGO, suggesting that intracellular MGO plays a role in protein localization [66]. A mis-localization of ZO-1 would lead to an overall reduced functionality of tight junction proteins.

While occludin and ZO-1 protein expression are unaffected by MGO, the same study found an increase in MGO-occludin glycation, thereby losing their functionality [66]. The increased MGO-adduct formation was prevented by co-treatment with NAC [66], suggesting the importance of predominantly intracellular glycation of MGO to occludin. Likewise, an in vivo study showed that increasing serum MGO by 1.5-fold in rats through intra peritoneal injection of MGO (2 mg/kg/day for 6 days), does not affect expression of tight junction proteins in brain endothelial cells [69]. However, whether the BBB permeability and the functionality of the tight junction proteins were altered, was not investigated in this study.

Moreover, long-term exposure to high glucose medium showed an increase of MGO-occludin adducts in BMECs and confirms that hyperglycemia-induced endogenous MGO formation plays a role in tight junction glycation [67]. In agreement, an increase in MGO-occludin adducts in the brain has also been demonstrated in diabetic mice [66, 67]. Thus, the effect of MGO on BBB permeability may be partly caused by the glycation of tight junction proteins by MGO.

### Detoxification of methylglyoxal in brain microvascular endothelial cells

Under physiological conditions, MGO is detoxified into D-lactate by the glyoxalase pathway (Fig. 2). The small yet abundant cellular antioxidant GSH [70] is a cofactor in the glyoxalase pathway [22]. It has been shown that manipulating GSH levels affects in vitro BMEC permeability [65, 66]. Increasing GSH levels with NAC, a GSH precursor, decreased endothelial permeability, whereas decreasing GSH levels with buthionine sulfoximine increased endothelial permeability [65, 66]. This shows the importance of GSH in maintaining BBB integrity and the effect may be mediated by the GSH-dependent glyoxalase activity. Moreover, MGO treatment was shown to significantly decrease the cellular GSH level and to only slightly decrease glutathione disulfide (GSSG) levels in BMEC cell lines [65, 66]. The subsequent decrease in GSH:GSSG ratio has consequences for MGO detoxification and the redox status [65], which may ultimately alter BBB permeability.

Furthermore, MGO treatment has been shown to increase S-glutathionylation of proteins (P-SSG) [65]. This post-translational protein modification, also known as thiol modification, can affect targeted proteins in response to reactive oxygen species (ROS) [71]. In endothelial cells, proteins prone to this thiol modification have been associated with consequences for inflammation, angiogenesis, and barrier function [72].

Moreover, pathological conditions such as inflammation, hyperglycemia and oxidative stress are known to reduce the expression of Glo1, the rate limiting enzyme in the glyoxalase pathway [22]. An in vitro study showed that under hyperglycemic condition, Glo2 but not Glo1 expression was reduced in BMECs [67]. Since GSH is a co-factor essential for the formation of S-D-lactoylglutathion by Glo1, and is released again in the formation of D-lactate by Glo2, a decrease in Glo2 activity might explain the reduced GSH availability.

### Methylglyoxal induces reactive oxygen species formation

The entanglement of the MGO detoxification pathway with the redox status, through affecting GSH availability and glyoxalase efficiency as explained prior, has implications for the ROS balance in BMECs exposed to MGO. Increased ROS formation leads to an increase in BBB permeability [66]. MGO itself has been shown to increase endogenous ROS formation [53, 57, 59, 61, 62, 64, 69] as well as lipid peroxidation by-products in BMECs [57, 59]. Additionally, ROS formation was shown to be inhibited by the MGO scavengers aminoguanidine [57, 61, 62] and edaravone [61].

It was shown that an MGO-induced increase in ROS levels can be partially counteracted by compounds with anti-oxidant properties such as retinoic acid [57] and edaravone [61, 62], thereby improving BMEC viability after MGO treatment. However, these compounds were able to reduce but not completely prevent the increasing permeability of BMECs caused by MGO treatment [57, 62]. Furthermore, while ROS scavengers were able to completely eliminate excess ROS levels caused by MGO treatment in BMECs, the increased barrier permeability by MGO treatment could not be completely restored [66]. Therefore, these studies indicate that MGO-induced ROS formation does play a role in MGO-induced BBB permeability, but that there are additional underlying mechanisms in MGO-induced BBB permeability.

### Mitochondrial dysfunction and apoptosis as consequence of elevated methylglyoxal

**Fig. 3:**
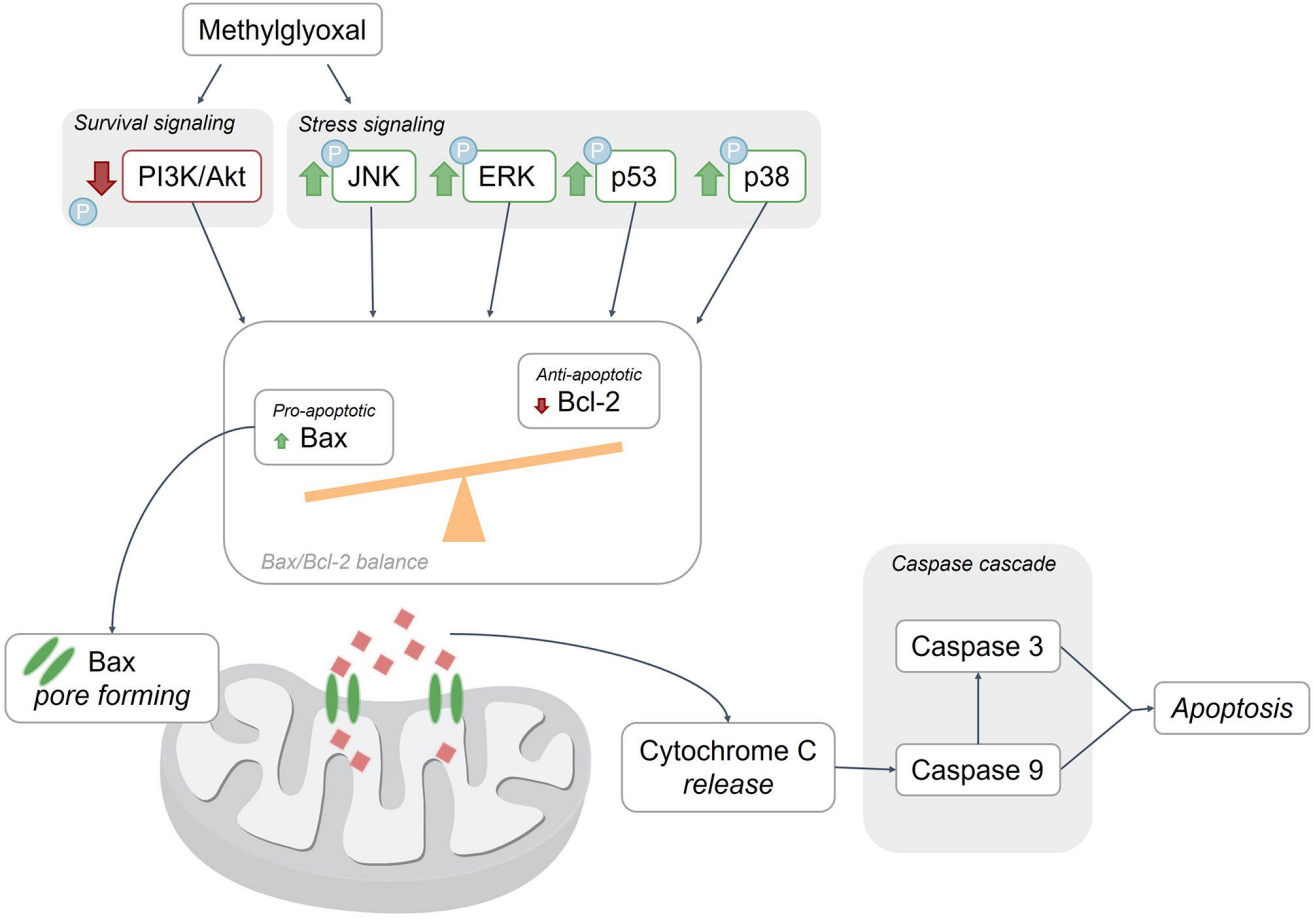
Apoptotic pathways after methylglyoxal exposure in brain microvascular endothelial cells. A decrease in cell survival signals and an increase in stress signaling by MGO treatment leads to an imbalance in Bax/Bcl-2, leading to Bax forming pores in the mitochondrial membrane [59, 64]. This leads in consequence to the release of cytochrome C from the mitochondria into the cytoplasm activating the caspase cascade [75] through caspase 9 and consequently also through caspase 3, leading to apoptosis [59, 64, 65]

MGO-associated ROS formation occurs primarily in mitochondria [53], and leads to the activation of apoptotic pathways in BMECs through at least three different pathways [58, 59, 61, 65] (Fig. 3). Firstly, treatment of BMECs with MGO leads to an activation of stress-associated pathways, characterized by an increase in phosphorylation of extracellular signal-regulated kinase (ERK) [59], c-Jun N-terminal kinase (JNK) [59, 64], and p38 [59], which are known responders to stress stimuli and affect endothelial cells by altering, for instance, barrier function, protein synthesis, and apoptosis [73]. Secondly, treatment of BMECs with MGO leads to a decrease in phosphorylated protein kinase B (Akt) to non-phosphorylated Akt, part of the phosphoinositide 3-kinases/Akt (PI3K/Akt) pathway [64, 74]. PI3K is known to be activated upon growth factor receptor signaling which leads to Akt phosphorylation, which overall improves cell survival and inhibits apoptosis [73]. Therefore, the observed downregulation of PI3K/Akt phosphorylation reflects a decrease in cell survival signaling. Last, treatment of BMECs with MGO induces an increase in pro-apoptotic tumor protein p53, leading to an imbalance in the Bax/Bcl-2 expression ratio with finally an increase in pro-apoptotic protein Bax [59, 64].

Through these pathways, MGO treatment decreases the membrane potential of BMEC mitochondria, reflecting a loss of mitochondrial integrity and functioning [65]. The increase in mitochondrial permeability is likely due to Bax protein upregulation, which forms pores in the outer membrane of mitochondria. Through these pores, pro-apoptotic cytochrome C is released into the cytoplasm and induces the caspase cascade [75]. Indeed, an increase in cytochrome C levels has been observed in MGO-treated BMECs [59]. In the MGO-induced caspase cascade in BMECs, a major role was observed for caspase 9 and caspase 3, but not caspase 8 [59, 64, 65] (Fig. 3). It has also been shown in other cell types that increased mitochondrial permeability can be directly caused by the glycation of MGO with mitochondrial proteins [22], however, there is no research currently supporting this in BMECs.

Additionally, a study found that the reduction in Akt phosphorylation leads to a reduced hypoxia-inducible factor 1 alpha (HIF-1α) [74]. Protein levels of HIF-1α are increased under hypoxic conditions to reduce oxygen demand and prevent cellular damage [76]. The decrease in HIF-1α as a response to MGO exposure, prevents an appropriate response to oxidative stress and enhances MGO-induced ROS production [74, 76].

MGO has also been reported to induce autophagic pathways [60] which may act as a protective mechanism in BMECs to maintain BBB integrity [77]. However, there is overlap in the autophagic and apoptotic pathways and increased autophagy can lead to apoptosis [78]. In fact, the rate of MGO-induced autophagy in BMECs first spikes, but then rapidly drops and the autophagic capabilities of the cell are exhausted [60]. This suggests that chronic exposure of BMECs to MGO might have more detrimental consequences for BBB integrity than a single spike of MGO.

Thus, MGO could lead to increased autophagy and/or apoptosis and thereby increase the BBB permeability in vitro [79]. To what extent apoptosis in BMECs by MGO can lead to BBB breakdown and BBB leakage in vivo remains, however, unclear.

### Signaling of the receptor for advanced glycation end-products

It has been shown that MGO treatment of BMECs could increase the expression of the receptor for AGEs (RAGE) [61]. RAGE is a multi-ligand receptor which binds predominantly pro-inflammatory associated proteins including AGEs, protein S100, β-amyloid and complement factor 3 A (C3A) [80]. Upon activation, nuclear factor kappa-light-chain-enhancer of activated B cells (NF-κB) is translocated to the nucleus where it alters gene expression, resulting in a pro-inflammatory state [80]. RAGE signaling in bEnd.3 cells (BMEC cell line), was found to be associated with decreased tight junction proteins expression levels [81–83] and an increase in immune cell adhesion through increased expression of vascular cell adhesion protein 1 (VCAM-1) [84, 85].

Furthermore, RAGE activation may downregulate Glo1 expression, reducing MGO detoxification, thus causing an increase in MGO-derived AGE formation and RAGE expression in a vicious cycle [80]. However, it is debated what the role of AGE-RAGE binding is, due to the multi-ligand properties of the receptor and since the in vivo plasma ratio of AGE to RAGE would suggest a saturation of RAGE. Therefore, treatment with AGEs or an increase in AGE formation would be unlikely to cause any increased in RAGE signaling [22]. However, to what extent MGO-derived AGEs are involved in the AGE/RAGE axis and what the consequences are for BBB permeability, is unknown.

## Methylglyoxal-induced functionality loss of mural cells

Mural cells of the brain microvasculature refer to the pericytes and VSMCs, which surround the BMECs [10]. These cells have a structural and regulatory function within the blood vessels and overlap in function and location with each other, sharing a wide range of subtypes with small phenotypic differences [10, 86–88]. The VSMCs are located more densely around the larger arterioles and venules (> 15 μm diameter) where cerebral blood flow is regulated through vasoconstriction and vasodilation upon demand through neurovascular coupling [87]. Pericytes are located towards, but not exclusive to, the capillary bed (< 10 μm diameter), where the cells play a role in the regulation of cerebral blood flow, BBB permeability, vascular stability, and angiogenesis [18, 87]. Some, but not all, subtypes of pericytes have contractile properties within the cerebral microvasculature like VSMCs [87–90]. The classification of different mural cells and their distinctive markers, location, and function have been thoroughly reviewed by Uemura and colleagues [86]. Beyond its impact on brain endothelial cells, MGO may also constitute a threat for BBB integrity via its impact on mural cells.

### Pericytes loss induced by methylglyoxal

Within the BBB, pericytes are located near endothelial cell junctions and at close proximity to astrocyte endfeet. The ratio of pericytes to endothelial cells is much higher in the cerebral microvasculature compared to the peripheral microvasculature [18]. The high number and location combined with their function render them important for the regulation of BBB function [18]. Pericyte dysfunction has been found to be associated with impaired brain perfusion [91, 92] and loss of BBB integrity [17, 91, 93].

Human brain microvascular pericytes incubated with high levels of glucose exhibited reduced proliferation and endothelial signaling with increased AGE levels [94]. Although the literature on the effect of MGO on brain microvascular pericytes is scarce, one publication suggests a role for endogenous formation of ROS in primary human microvascular pericytes after in vitro MGO treatment [95]. More literature is, however, available on retinal pericytes. MGO treatment of retinal pericytes in vitro also leads to endogenous ROS formation, which in turn leads to a reduction in viability, and an increase in caspase 3-mediated apoptosis [96–98]. In human primary retinal pericytes, MGO inhibits Glo1 activity [99], which could be the cause of an increase in ROS formation.

Moreover, MGO treatment of cultured retinal pericytes led to an enhanced NF-κB translocation to the nucleus [96, 98]. Consequently, this was accompanied by an increase in expression of inducible nitric oxide synthase (iNOS) protein [96]. Inhibiting iNOS directly or indirectly through inhibiting NF-κB in MGO-treated primary pericytes, showed an improvement of cell viability [96]. Nitric oxide (NO) appears to play a major role in the viability of pericytes when exposed to MGO since iNOS inhibition improved cell viability without changing NF-κB DNA binding activity [96]. Under physiological conditions, NO signaling in pericytes causes vasodilation [18], however, chronically increased NO levels result in nitrative stress, which in turn is known to play a role in neurodegenerative disease and diabetic neuropathy [100].

In vivo evidence for the effect of MGO on pericytes and the consequence for BBB integrity is limited. One study showed a loss of retinal pericytes after four weeks of treatment with MGO in drinking water [101]. Additionally, there are several studies showing the effect of diabetes on pericyte number and coverage. Pericyte loss was found in both type 1 [102] and type 2 diabetes [94, 102, 103]. However, in a genetic model of type 1 diabetes (Akita), BBB dysfunction was not associated with pericyte loss [104], which challenges the role of MGO in diabetic pericyte loss. Whether pericyte loss is caused directly by MGO formation in diabetes, remains to be investigated.

### Methylglyoxal reduces contractility of vascular smooth muscle cells

Dysfunction of VSMCs has been associated with loss of BBB integrity and neurodegenerative disease [13, 105]. The VSMCs in the brain are responsible for regulating cerebral blood flow by rapidly adjusting arterial diameter, via contraction or dilation of arterioles, based on neuronal demand [87]. Sufficient perfusion of the brain mediated by VSMCs-mediated dilation of arterioles and the stabilizing properties of VSMCs within the BBB, are important to maintain a healthy BBB [105].

In vitro treatment of human brain VSMCs with MGO showed a reduction in cell viability, although, VSMCs appear to be more resilient to MGO than human brain endothelial cells [53]. In cultured femoral VSMCs, a high concentration MGO treatment reduces DNA synthesis, cell viability, cell migration, and MGO-glycation of platelet-derived growth factor receptor β (PDGFRβ) leading to reduced PDGFRβ signaling [106]. Platelet-derived growth factor (PDGF), a PDGFRβ ligand, is excreted by endothelial cells and binds to the PDGFRβ located on mural cells. PDGFRβ signaling is crucial for the survival and proliferation of mural cells, which, in turn, play a vital role in the survival, proliferation, and stability of endothelial cells [107].

In the cerebral vasculature, PDGFRβ signaling also plays an important role in vascular stability and BBB integrity [94]. PDGFRβ coverage was shown to be reduced in the cerebral vasculature of diabetic rats leading to a reduced vascular density and loss of BBB integrity [94]. Whether diabetes associated increased levels of MGO are directly responsible for BBB integrity loss through reduced PDGFRβ signaling in vivo remains to be determined.

Direct in vivo superficial application of MGO on the rat brain through a cranial window, reduced vasodilation by a change in endothelial cell signaling rather than VSMC signaling [53]. However, increasing MGO detoxification through Glo1 overexpression in VSMCs through viral transfection improves cerebral vascular reactivity and arterial perfusion in diabetic rats [53]. This emphasizes the importance of adequate MGO detoxification by VSMCs in order to maintain a normal vascular reactivity in endothelial cells. Furthermore, Glo1 overexpression in VSMCs also reduces BBB leakages in diabetic rats showing that MGO in VSMCs are important for BBB integrity [53].

Moreover, it is important to address the role of hypertension and the consequence for BBB integrity because in angiotensin II mediated hypertension Glo1 protein expression was reduced in brain microvessels [108]. By increasing Glo1 expression in vivo through viral transfection, VSMC proliferation was reduced and hypertension mediated cerebrovascular remodeling could be prevented [108]. Thus, increased levels of MGO and reduced Glo1 activity shown to play a role in hypertension [13, 22].

Taken together, MGO has an effect on both hypertension and BBB integrity. Hypertension can negatively affect BBB integrity, and it is known that loss of BBB integrity can accelerate the development of hypertension, leading to a vicious cycle [13].

## Resilience of astrocytes to methylglyoxal

Astrocytes are glial cells with a wide range of phenotypes linked to specialized functions in the central nervous system. Astrocytes play an important role in the neurovascular crosstalk, support of neuronal functioning and maintenance of homeostasis within the brain parenchyma [109]. Within the neurovascular unit, astrocytes harbor several key functions. Firstly, astrocytes play an important role in regulation of nutrients and glucose transport into the brain parenchyma by modulating the BBB permeability [16]. Secondly, astrocyte endfeet form the perivascular space which plays a role in metabolic waste clearance [19]. Lastly, astrocytes play an important role in neurovascular coupling, connecting neurons and the vasculature [110].

### Astrocyte-neuron metabolic interplay

Astrocytes play an important role in brain glucose metabolism through their ability to modulate glucose transporter expression in endothelial cells and provide the appropriate amount of energy to neurons [16, 111]. In fact, astrocytes metabolize more glucose than required for their own energy supply, leading to an increase in lactate, the preferred energy source of neurons. This metabolic interplay is also known as the neuron-lactate shuttle [111].

In accordance with the fact that astrocytes metabolize relatively large amounts of glucose, a study of human post-mortem brain tissue showed that MGO-derived AGEs were predominantly present in astrocytes and not in neurons or microglia [112]. Astrocytes have to cope with large amounts of endogenously formed MGO and, like other cell types, astrocytes are able to detoxify MGO through the glyoxalase pathway [113]. In fact, Glo1 is strongly co-localized with astrocytic markers in the mouse cerebral cortex and primary astrocytes and in vitro astrocytes are very efficient in the detoxification [111]. While astrocytes are metabolically flexible and appear resilient to MGO, in vitro studies show that MGO reduces cell viability in a dose dependent manner [111, 114–117].

In vitro treatment of astrocytes with MGO leads to a dose dependent increase in Glo1 [117] and blocking Glo1 expression with short interfering RNA (siRNA) reduces the overall viability of cultured astrocytes [111]. Thus, correct astrocyte functioning is essential for the detoxification of MGO in the brain and, thereby, is essential for neuronal cell survival [111, 113]. The exposure of astrocytes to MGO (2.5mM) initially causes a decrease in intracellular GSH availability, which returned to baseline levels over time (24 h) [111]. This was, however, not achieved upon treatment of astrocytes with higher MGO concentrations (≥ 3.5mM) after 24 h [111, 117]. Additionally, lower MGO concentrations (≤ 2.0mM) increases intracellular GSH after 24 h [111]. The depletion of the GSH store is believed to be due to the activity of Glo2, which is observed to be slower than Glo1 in astrocytes, thereby limiting the detoxification rate [111].

GSH can be reduced from GSSG by GSH reductase (GR) which requires formation of nicotinamide adenine dinucleotide phosphate (NADP+) from dihydronicotinamide adenine dinucleotide phosphate (NADPH) [111]. It was shown that with a moderate MGO dose (2.5mM), NADP + levels do not change, however, NADPH levels increased after 30 min [111]. The stability of NADP + levels and the increase in NADPH levels over time indicate that astrocytes have an effective compensatory mechanism for sufficient GSH store replenishing. These findings too show that astrocytes are metabolically flexible and are essential for the detoxification of MGO in the brain [111, 113]. However, studies investigating MGO in astrocytes have only investigated the detoxification in relation to neurons. Therefore, astrocytic detoxification of MGO in relation to BMECs and the effect on the BBB should be further investigated.

### Methylglyoxal reduces insulin signaling in astrocytes

Insulin signaling in astrocytes is essential for maintaining proper cellular function [118]. MGO treatment in vitro leads to a downregulation of the insulin signaling pathway in primary astrocytes, through increased phosphorylation of the insulin receptor and insulin receptor substrate [115]. This, leads to a decrease in Akt phosphorylation downstream which causes an activation of apoptosis associated pathways of caspase 3 and caspase 7 [115]. The activation of caspases leads to the cleavage of poly ADP ribose polymerase (PARP), which induces apoptosis [115]. Astrocyte co-treatment with insulin reduces MGO-associated apoptosis [115], indicating an important role of Akt signaling pathway in MGO induced loss in viability and apoptosis.

In a model of insulin deficiency in mice, there was BBB damage and a retraction of astrocytes at the BBB, possibly due to reduced insulin signaling [119]. Furthermore, reduced insulin signaling in astrocytes leads to an altered astrocyte morphology, reduced mitochondrial function, and altered mitochondrial function [118]. It could therefore be speculated that MGO-associated reduced insulin signaling might affect viability and morphology of astrocytes in close proximity to the cerebral vasculature, leading to a remodeling of the perivascular space and a loss of neurovascular coupling. The interaction between MGO, astrocytic insulin signaling and BBB would need to be further investigated.

### Methylglyoxal induced astrocyte activation and inflammation

Activation of astrocytes, also known as astrogliosis, plays a large role in brain repair and scar formation as a response to neuronal injury and inflammation [120]. Astrogliosis has a neuroprotective role and also has the ability to repair the BBB when compromised. However, on the long term or under specific pathological conditions, it can have detrimental effects on the brain and the BBB [120].

MGO causes an inflammatory-like response in astrocytes in vitro [115, 116]. Over time, MGO first leads to an increased activation of c-Jun protein through JNK phosphorylation, which then lead to pro-inflammatory cytokine production [115]. Ultimately, the inflammatory response is marked by an increase in glial fibrillary acidic protein (GFAP) and protein S100B [115, 116]. GFAP is a highly expressed astrocytic marker and an increase of GFAP expression is associated with the neuroprotective function of astrocytes [120]. Protein S100B, a calcium binding protein predominantly but not exclusively expressed by astrocytes, is expressed in low levels by astrocytes during neuroprotective astrogliosis and further increased in destructive astrogliosis and can therefore be used as a marker for neuronal injury in BBB disruption [121, 122].

In hippocampal slices (ex vivo) it was also shown that MGO increased S100B secretion [123, 124]. Moreover, co-treatment of hippocampal slices with aminoguanidine did not influence S100B levels suggesting that the impact of MGO on S100B in vitro and ex vivo is moderate, and indicates a healthy neuroprotective response of astrocytes to the increase of MGO in the cell environment.

In vivo, it was shown that daily intraperitoneal injection with very high amounts of MGO (60 mg/kg) in mice for 6 weeks, led to an increase inflammatory response in the hippocampus [116]. It can, however, be debated whether this inflammatory response in the hippocampus is due to the effect of MGO on astrocytes, whether it is an effect of MGO on the brain and BBB, or whether it is an indirect effect of the glycating potency of MGO elsewhere in the body leading to systemic inflammation and impairing affecting other organs besides the brain.

Intracerebroventricular (ICV) injection of MGO (15μmol) in rats was shown to lead to an increase in IL-1β but a reduction of S100B gene expression, and was associated with reduced cognitive function [125, 126]. The reduced cognitive function coincided with a reduced expression of water channel aquaporin 4, a marker for astrocyte endfeet, and an increase in serum albumin in the cerebrospinal fluid (CSF) [126]. This suggests that increasing MGO in the CSF leads to an increased permeability of either the BBB or the blood-CSF barrier [5]. It is, however, unclear how increased MGO levels in the CSF influences the barriers’ permeability.

## Microglia, producers of methylglyoxal

Microglia, so-called brain resident macrophages, are the innate immune cells of the brain and play a role in clearance of cellular debris, synaptic pruning, and defense against pathogens, thereby exerting a homeostatic function in the brain [4]. Microglia are present in close proximity to brain microvessels where they continuously survey their surrounding for potential pathogens. Upon activation, microglia produce pro-inflammatory cytokines which can increase permeability of the BBB, allowing passage of immune cells into the brain parenchyma [15]. Furthermore, there is evidence that microglia are involved in regulating cerebral blood flow by interacting with the brain microvasculature [15]. Correct microglial functioning is therefore essential to maintain BBB integrity and protect the brain against injuries. A recent review has described the importance of MGO formation and utilization in different immune cells [127].

In normal appearing white matter in post mortem brain tissue, MGO-derived AGEs and the microglial marker allograft inflammatory factor 1 (Aif1/Iba1) were not co-localized [112]. This suggests that in microglia with an anti-inflammatory phenotype, there is limited MGO formation and/or efficient MGO detoxification. On the other hand, stimulation of microglia (N11 cell line) with interferon-γ (IFN-γ) and lipopolysaccharide (LPS), leads to MGO formation and release into the environment [128]. An increased MGO formation during microglial activation may be caused by a metabolic shift since microglial cells use the glycolysis pathway as a main source of energy upon activation, which could explain the lack of MGO-derived AGEs in resting microglia [129].

Apart from the formation of MGO during glycolysis in microglia, MGO can also shift microglia towards a pro-inflammatory phenotype. In vivo studies have shown that exogenous application of MGO, either through drinking water or intravitreal injection, leads to an increase in expression of cluster of differentiation 74 (CD74) protein in the retina [101, 130]. CD74 is a marker for pro-inflammatory microglia and is also found to be upregulated in diabetic retinopathy [130]. It might be that the effect of MGO on microglial phenotype is an indirect effect through AGE-RAGE signaling. It has been shown that RAGE is highly co-localized with Iba1 positive microglia [112] and AGE-RAGE signaling is known to play a large role in the activation of microglial cells [51].

Additionally, in diabetic rats, the increase in the detoxification of MGO by the overexpression of Glo1 in cerebral VSMCs, also reduces MGO-derived AGEs and the number of Iba1 positive microglia [53]. This indicates that reduction of MGO and MGO-derived AGEs in microglia reduces the microglial pro-inflammatory state in vivo. However, whether these in vivo effects are directly caused by MGO or MGO associated BBB integrity loss and damage in other cell types is unsure.

As the interplay between brain immune cells and brain microvessels is gaining importance for cerebral small vessel disease [131–133], it becomes urgent to understand the effect of MGO on microglia/brain immune cells and how this might affect the BBB integrity. The very limited findings on MGO and microglia should encourage studies to investigate possible differential effects between intracellular and extracellular MGO. Furthermore, it is important to consider the vast heterogeneity of microglial phenotypes throughout different brain areas [134, 135], but also large cellular heterogeneity between all brain immune cells [4].

## Concluding remarks

We reviewed the literature on the effects of MGO on the different cell type of the BBB. Emerging evidence based on experimental research indicates that MGO may lead to cell damage or cell death, resulting in BBB integrity loss (Fig. 1). However, the translation of these findings remains challenging, because the majority of the studies done so far were performed with a high exogenous concentration of MGO ranging from 100μM to over 50mM. Considering that plasma MGO concentrations measured in humans is ~ 250nM [136], and 10-100nM in CSF [112, 137], the physiological relevance of a 1000 to 10,000 times higher concentration, as used in most of the in vitro work, can be questioned. Additionally, MGO levels in brain could range from 25ng/mg protein to 300ng/mg protein in rats, or 500nmol per mmol lysine in humans [53, 112]. However, most studies reporting the effect of MGO on the brain, do not measure or report MGO concentration, or report the measured quantity in different ways, which makes comparison of tissue MGO-content in literature challenging.

Moreover, most of the studies have used a commercially available batch of MGO, which is known to be contaminated [138]. We cannot exclude the possibility that negative effects described in literature are due to contaminants, or a combined effect of contaminants with MGO. Thus, the use of high concentrations of commercially available MGO should be avoided.

Furthermore, the method of applying or increasing of MGO in experimental studies should be taken into consideration. When looking into the effect of MGO as by-product of glycolysis, it is important to use the appropriate model to increase endogenous MGO formation through either increasing glycolysis or reducing detoxification by reducing Glo1 activity. Interestingly, we recently found in humans, that a higher habitual intake of MGO is associated with an increase in plasma MGO [136] and with beneficial, rather than negative effects on low grade inflammation [139]. In the field of cancer research, MGO has been described to have a hormetic effect, showing different effects depending on the concentration [140]. The beneficial effect at low concentration of extracellular MGO could be ascribed to possible antioxidant properties of MGO in low concentrations through the KEAP1-Nrf2 pathway [44, 139].

## Conclusion

While the effect of MGO on the BBB as a multicellular system remains to be further elucidated, MGO is likely to have the most predominant effect on endothelial cells and mural cells, since these are in closest contact with the circulation, take up glucose in an insulin-independent manner and thus would be most prone to spikes of glucose and the consequent MGO formation. Astrocytes on the other hand, are less likely to be strongly affected by acute increased levels of MGO or increased MGO formation, due to its effective detoxification system. However, when chronically exposed to elevated MGO, astrocytes might reduce their detoxification properties leading to overall damage to the cell and its environment. Furthermore, during chronic hyperglycemia, the brain will reduce glucose uptake, preventing toxic glucose levels and consequent MGO formation in the brain parenchyma [23]. Based on available literature, microglia are unlikely affected directly, but could shift to a pro-inflammatory state upon RAGE activation by MGO-derived AGEs, or when BBB integrity is lost. In fact, microglia might increase MGO formation upon activation, which might be beneficial for the inflammatory state in immune cells [127]. What would happen to astrocytes and microglia as a response to increased MGO if the BBB is already lost, for instance during neuroinflammation, is unclear. Although the effects of MGO on the single cell types of the BBB are clear, it should be emphasized that in vitro experiments on these cell types separately, are not a complete representation of the BBB system due to the strong interplay of the different cell types present [141, 142].

In summary, there is a strong set of data indicating the negative effects of MGO on the BBB integrity. Because of the complexity of the BBB as a multicellular system, additional research is necessary to elucidate the precise mechanism through which MGO affects the BBB as a whole, taking into consideration appropriate methodological approaches.

## Data Availability

Not applicable.

## Abbreviations

AGEs: Advanced glycation end-products
Aif1/Iba1: Allograft inflammatory factor 1
AKR: Aldo-keto reductase
Akt: Protein kinase B
ALDH: Aldehyde dehydrogenase
Arg-Pyr: Nδ-(5-hydroxy-4,6-dimethylpyrimidine-2-yl)-L-ornithine argpyrimidine
BBB: Blood-brain barrier
BMECs: Brain microvascular endothelial cells
C3A: Complement factor 3 A
CD74: Cluster of differentiation 74
CEdG: N^2^-carboxyethyl-2’-deoxyguanosine
CEL: N^ε^-(1-carboxyethyl)lysine (CEL)
CSF: Cerebrospinal fluid
DHAP: Dihydroxyacetone phosphate
ERK: Extracellular signal-regulated kinase
G3P: Glyceraldehyde-3-phosphate
GFAP: Glial fibrillary acidic protein
Glo1: Glyoxalase 1
Glo2: Glyoxalase2
GSH: Glutathione
GSSG: Glutathione disulfide
HIF-1α: Hypoxia-inducible factor 1 alpha
IFNγ: Interferon gamma
iNOS: Inducible nitric oxide synthase
JNK: C-Jun N-terminal kinase
KEAP1: Kelch-like ECH associated protein 1
LPS: Lipopolysaccharide
MG-H: Hydroimidazolones
MGdG: 3-(2’-deoxyribosyl-6,7-dihydro-6,7-dihydroxy-6/7-methylimidazo-[2,3-b]purin-9(8)one
MGO: Methylglyoxal
MICA: Mercaptomethylimidazole crosslinks between arginine and cysteine
MODIC: 2-ammonio-6-((2-[(4-ammonio-5-oxido-5-oxopentyl)amino]-4-methyl-4,5-dihydro-1 H-imidazol-5-ylidene)amino)hexanoate
MOLD: 1,3-di(N^ε^-lysino)-4-methl-imidazolium (MOLD)
NAC: N-acetyl cysteine
NADP+: Nicotinamide adenine dinucleotide phosphate
NADPH: Dihydronicotinamide adenine dinucleotide phosphate
NF-κB: Nuclear factor kappa-light-chain-enhancer of activated B cells
NO: Nitric oxide
Nrf2: Nuclear factor erythroid 2-related factor 2
P-SSG: S-glutathionylation of proteins
PDGF: Platelet-derived growth factor
PDGFRβ: Platelet-derived growth factor receptor beta
PI3K: Phosphoinositide 3-kinases
RAGE: Receptor for advanced glycation end-products
ROS: Reactive oxygen species
siRNA: Short interfering RNA
TEER: Trans-endothelial electrical resistance
THP: Nδ-(4-carboxy-4,6-dimethyl-5,6-dihydroxyt-1,4,5,6-tetrahydropyrimine-2-yl)-L-ornithine
VCAM-1: Vascular cell adhesion protein 1
VSMCs: Vascular smooth muscle cells
ZO-1: zonula occludens 1
